# OD07 Disaggregation Of The Costs Of Pharmaceutical Research And Development

**DOI:** 10.1017/S0266462324001430

**Published:** 2025-01-07

**Authors:** Daniel Fabian, Claudia Wild

## Abstract

**Introduction:**

Costs for pharmaceutical products are increasing. Pharmaceutical companies claim that high research and development (R&D) costs are the reason for the steep price increase of new products. However, there exists little data to support such claims; there is a lack of transparency in R&D cost reporting. This research intends to analyze and to disaggregate the costs of pharmaceutical R&D.

**Methods:**

Studies on the costs of introducing new medications to the market can differ substantially in their methodology, their origin of data, and their results. A scoping review was conducted on costs of R&D for pharmaceutical products using Embase, PubMed, and EconLit, using a combination of the terms “drug research and development” and “costs” or “drug research and development” and “expenditure.” Additionally, semi structured interviews with 16 experts from non-governmental organizations (NGOs), pharmaceutical companies, academic researchers, and not-for-profit pharmaceutical companies were conducted to identify the main driving factors for rising costs of new drugs.

**Results:**

Out of 24 studies that analyzed mixed therapeutic fields, the five highest cost estimates had affiliations with industry or received funding from pharmaceutical companies. Non-affiliated researchers are unable to reproduce studies that use confidential data and therefore cannot check the validity of the results. Additionally, different definitions for R&D make analyzing costs challenging. The interviewees emphasized that driving factors influencing costs for pharmaceutical R&D are therapeutic indication, drug complexity, number of patients in clinical trials, length of the development process, and attrition rates.

**Conclusions:**

Due to the diverse nature of drug development and the confidential information held by pharmaceutical companies, it is a challenge to provide an exact assessment of the average costs of pharmaceutical R&D. Transparency policies with well-defined definitions for R&D are necessary to level the information asymmetries between the private and the public at price negotiations.

